# Guided nonlinear optics for information processing

**DOI:** 10.1515/nanoph-2025-0348

**Published:** 2025-08-05

**Authors:** Daniel Brunner, Birgit Stiller, Demetri Psaltis

**Affiliations:** Université Marie et Louis Pasteur, CNRS UMR 6174, Institut FEMTO-ST, 25000, Besançon, France; Max-Planck-Institute for the Science of Light, Staudtstr. 2, 91058 Erlangen, Germany; Department of Physics, Friedrich-Alexander-Universität Erlangen-Nürnberg, Staudtstr. 7, 91058 Erlangen, Germany; Institute of Photonics, Leibniz University Hannover, Welfengarten 1A, 30167 Hannover, Germany; EPFL, Institute of Electrical and Micro Engineering, 1015 Lausanne, Switzerland

This special issue explores the dynamic and rapidly evolving field of optical computing, with a focus on guided nonlinear optics for information processing. Recent years have seen a resurgence of interest in this area [1], fuelled by ground-breaking experimental demonstrations of advanced computational capabilities and the development of sophisticated photonic devices. A key example of the synergy between optical computing and nonlinear guided propagation is the creation of novel hardware, utilising the unique properties of light when confined and guided in nonlinear optical materials.

In this issue, we feature current trends within the community, highlighting how guided nonlinear optics provide a robust hardware foundation that aligns with the demands of modern computational paradigms [2]. The intersection of optical computing and nonlinear guided propagation is a cornerstone of contemporary optics research [3]. Photonics offers numerous advantages in this context, including the potential for massive parallelism, ultra-fast operation speeds, and potentially a significantly reduced power consumption. Furthermore, photonic architectures present compelling opportunities for performing computations that transcend the capabilities of current hardware, fostering a uniquely reciprocal relationship between these two domains.

Slinkov et al. experimentally demonstrate a novel approach to implement a variety of activation functions by using the interaction of light and sound via a double-Brillouin-amplifier setup featuring frequency-selectivity, all-optical control and preservation of the optical input coherence [4]. Kesgin and Teğin present an experimental and theoretical study on multimode interaction in optical fibres operated near spatiotemporal chaos, demonstrating they could demonstrate that data classification can be enhanced close to the chaotic edge for different use cases such as the classification of Breast MNIST, Fashion MNIST, and EuroSAT datasets [5]. Hary et al. [6] as well as Saeed et al. [7] establish a link between the fundamental properties in nonlinear fiber propagation and task-independent metrics such a dimensionality, consistency and nonlinearity to gauge such system’s computational capacity, while additionally benchmarking their performance in popular classification data-sets. Manuylovich et al. [8] expand the concept of extreme learning machines in nonlinear photonic systems via a trainable input encoding mask to effectively increase the representational capacity of the feature space. Using fiber-optical components, Rübeling et al. [9] realize programmable photonic frequency optical neural networks that feature *in situ* training. Finally, Oguz et al. [10] use a digital twin, i.e. a neural model that differentiably approximates the optical system for training an optical neural network realized via ultrashort pulses propagating in multimode fibres back propagation to achieve state-of-the-art image classification accuracies in experiments.

Beyond the implementation of computational concepts leveraging nonlinear optics, the special issue furthermore makes use of modern computational concepts for the design and modelling of nonlinear photonic integration. Shao et al. [11] leverage physics-inspired deep learning to enable an efficient, reliable, and flexible paradigm of inverse design of nonlinear metasurfaces. Alexis et al. [12] extend temporal reflection and refraction analogies from the case of singlemode optical fibres to multimode fibres, showing that nonlinear multimode fibers provide novel degrees of freedom that permit control over optical pulse interactions; while Sader et al. [13] investigate noise-driven modulation instability during nonlinear fiber propagation, demonstrating the potential of coherent optical seeding and machine learning to jointly control incoherent spectral broadening dynamics. Finally, Finot and Rochette reflect on numerous challenges and successes associated with the Mamyshev optical regenerator, which has proven highly valuable in the domain of high-power fiber lasers and exhibits compatibility with optical chip integration [14].
